# Facial Expression–Based Evaluation of the Emotion Estimation Software Kokoro Sensor in Healthy Individuals: Validation and Reliability Pilot Study

**DOI:** 10.2196/81868

**Published:** 2026-02-26

**Authors:** Shota Yoshihara, Satoru Amano, Kayoko Takahashi

**Affiliations:** 1 Department of Rehabilitation Sciences, Kitasato University Graduate School of Medical Sciences, Kanagawa, Japan; 2 School of Allied Health Science, Kitasato University, 1-15-1, Kitasato, Minami-ku, Sagamihara, Kanagawa, 252-0373, Japan, 81 042 778 9849

**Keywords:** emotion detection technology, facial expressions, artificial intelligence, AI, emotion AI, rehabilitation

## Abstract

**Background:**

In recent years, artificial intelligence (AI) systems have increasingly been used to assess emotional states in health care. AI offers a safe, quick, user-friendly, and objective emotional evaluation method. However, evidence supporting its implementation in health care remains limited.

**Objective:**

This study aimed to explore the concurrent validity and test-retest reliability of emotion recognition AI based on facial expressions.

**Methods:**

In this study, we used the Kokoro Sensor, an accurate and widely recognized automated facial expression recognition system. The Japanese version of the Profile of Mood States–Short Form was used to screen the potential influence of mental states on facial expressions. The study participants made positive, negative, and neutral expressions, which were analyzed by the emotion recognition AI. Agreement between the results of the AI and subjective evaluations was assessed by participants and a researcher using a 4-point Likert-type scale. The facial expressions and emotion analysis process were repeated after a 30-minute interval to investigate reliability. Concurrent validity was evaluated using the content validity index (CVI) and κ coefficient, and test-retest reliability was determined using the κ coefficient.

**Results:**

The study participants were 40 individuals whose mental states did not deviate from the reference range of the Profile of Mood States manual. Among the participants, the CVI values for positive, neutral, and negative expressions were 95%, 98%, and 85%, respectively. Among the researchers, the corresponding CVI values were 100%, 100%, and 70%, respectively. The overall weighted κ coefficient was 0.55 (CI 0.44‐0.67), indicating moderate agreement. The agreement was almost perfect for distinguishing positive from neutral expressions (κ=0.83, 95% CI 0.70‐0.95) but not statistically significant for distinguishing negative from neutral expressions (κ=0.15, 95% CI –0.07 to 0.37). Test-retest reliability analysis showed an overall weighted κ coefficient of 0.66, reflecting substantial reliability. Almost perfect agreement was observed for distinguishing positive from neutral expressions (κ=0.85, 95% CI 0.73‐0.97), while distinguishing negative from neutral expressions showed limited reliability (κ=0.36, 95% CI 0.16‐0.57).

**Conclusions:**

Our findings suggest that the Kokoro Sensor may be useful for identifying positive affect, given its acceptable concurrent validity for overall valence estimation and its high agreement for distinguishing positive from neutral expressions. However, concurrent validity for negative expressions did not meet the prespecified benchmark based on the researcher’s ratings, and agreement for distinguishing negative from neutral expressions was limited, which may constrain clinical utility for detecting negative affect. Therefore, in clinical settings, the Kokoro Sensor should be used as an assistive tool rather than a stand-alone method.

## Introduction

In recent years, artificial intelligence (AI) systems designed to predict human emotional states have garnered significant attention, especially in health care settings. These emotion recognition technologies have been the focus of intense research [1,2], using techniques such as facial recognition [3], speech analysis [4], text processing [5], and electroencephalography-based brain activity monitoring [6]. In health care settings, AI-driven real-time emotion recognition holds substantial promise, enabling providers to assess psychological states, such as pain and anxiety, and develop more personalized treatment plans swiftly. This technology addresses the limitations of traditional methods, which often depend on subjective patient self-reports, interviews, or clinician observations.

One of the most established methods for emotion recognition is the facial action coding system (FACS), a technique renowned for its precision in facial expression analysis [7-11]. However, FACS is a human-driven method that requires not only substantial time for facial expression classification but also extensive training to acquire the necessary specialized skills [12]. Consequently, its practical applicability in fast-paced clinical settings, where timely responses are essential, is limited. Accordingly, automated and efficient AI solutions that can be seamlessly integrated into clinical workflows are needed.

The Kokoro Sensor (CAC Inc) is a commercially available AI system for automated facial expression analysis that identifies 21 facial expressions and 7 basic emotions and outputs probability-based scores (0‐100) along with valence labels (positive, neutral, and negative) using algorithms grounded in Ekman basic emotion theory and FACS [7-11]. According to publicly available documentation [13], the underlying deep learning models were trained and tested on a corpus exceeding 14 million videos from 90 countries, providing substantial geographic diversity. Although detailed demographic composition is not disclosed, its size and international coverage are presumed to support model robustness and facilitate cross-cultural generalizability.

However, despite its use in a clinical setting [14-16], the validity and reliability of the Kokoro Sensor for applications involving health care populations remain largely unexamined.

For an AI emotion tool to achieve clinical credibility, psychometric performance—particularly validity and reliability—should be established in line with the Consensus-based Standards for the Selection of Health Measurement Instruments (COSMIN) framework [17]. Moreover, the evaluation should account for factors that can influence facial expressions across populations, such as cross-cultural variation in display rules (eg, between Western and Eastern populations) [18], individual variability and population-level anatomical differences in facial musculature [19-23], and the greater suppression of facial movements reported in Eastern cohorts [24]. Given these considerations, a focus on valence (positive or neutral or negative), rather than fine-grained discrete emotions, may offer a more robust and reproducible target across diverse groups.

To address this gap, this pilot study evaluated the Kokoro Sensor’s (1) concurrent validity—agreement between its valence outputs and human ratings—and (2) test-retest reliability in healthy young Japanese adults. Establishing these properties provides evidence for the potential adjunctive use of this in clinical assessment.

## Methods

### Participants and Eligibility

Between February and June 2024, participants were recruited via posters; interested individuals contacted the first author (SY) either by email or in person. The first author coordinated enrollment, provided study information, and obtained written informed consent, and either SY or SA was present at all experimental sessions.

Eligible participants were Japanese adults aged 18‐30 years who were able to attend in-person laboratory sessions. The exclusion criteria were as follows: (1) a history of, or current, facial neuromuscular disorder; (2) a diagnosed psychiatric disorder; (3) insomnia; and (4) self-reported current treatment for, or current symptoms consistent with, a sleep disorder, a stress-related condition, or fatigue, assessed via a brief self-report screening conducted verbally at enrollment; or (5) a Japanese Profile of Mood States–Short Form (POMS-SF) Total Mood Disturbance (TMD) T-score of 70 or higher. These exclusion criteria were based on self-report and were not verified by clinical diagnosis or standardized screening thresholds.

### Mood Assessment

Current mood state was evaluated using the POMS-SF questionnaire [25]. The POMS-SF indexes transient mood across 7 subscales: Anger-Hostility, Confusion-Bewilderment, Depression-Dejection, Fatigue-Inertia, Tension-Anxiety, Vigor-Activity, and Friendliness. TMD was calculated as the sum of negative subscales (Anger-Hostility, Confusion-Bewilderment, Depression-Dejection, Fatigue-Inertia, and Tension-Anxiety) minus the sum of positive subscales (Vigor-Activity, Friendliness), with higher scores indicating greater mood disturbance.

POMS-SF scores were standardized by sex and age in accordance with the guidelines outlined in the POMS-SF manual [26]. We prespecified TMD 70 or higher as an exclusion threshold to avoid testing during periods of marked negative mood, which can blunt positive facial expressivity. By contrast, elevated positive-mood scores do not indicate affective distress and generally do not preclude the ability to produce instructed negative expressions; therefore, they were not used as exclusion criteria.

### Sample Size

In this study, the required sample size for calculating the weighted κ coefficient, the primary analysis, was estimated based on a previous study [27]. The estimation was conducted under the assumption of a 2-sided significance level of .05 and a statistical power of 0.8. Given a planned weighted κ coefficient of 0.8 for a 3×3 contingency table, the sample size necessary to achieve the desired precision was calculated to be 39 participants. To mitigate the potential impact of participant attrition and unusable data, the final target sample size was adjusted to 40 participants.

### Study Setting

This cross-sectional study was conducted between March and June 2024 using a structured experimental design. Standardized equipment, including a high-resolution web camera (HD Webcam Meet, model number C960; EMEET), which was externally mounted on top of a personal computer, was used across all conditions. All experiments were carried out in a controlled, quiet laboratory environment.

### Study Flow and Procedure

**Figure 1.**
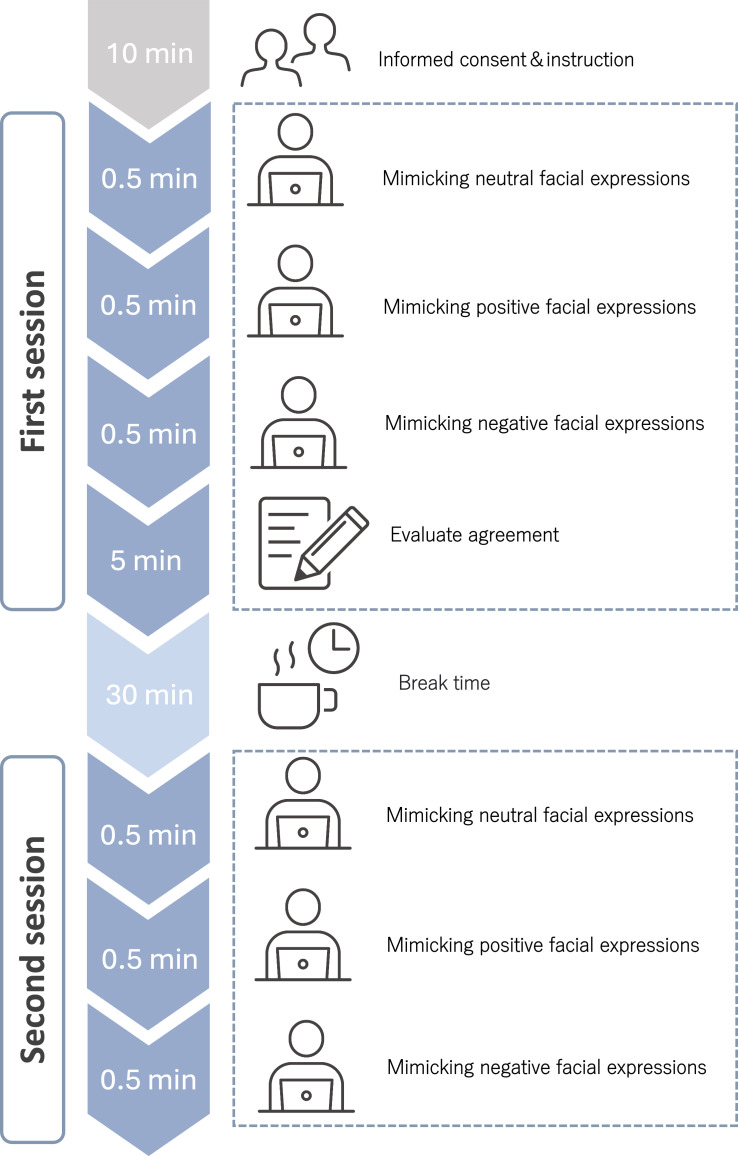
Overall experimental flow.

Figure 1 shows the overall experimental flow. This study followed a structured experimental design, with a total time lasting approximately 50 minutes. This included 5 minutes for obtaining informed consent regarding the video recording for facial analysis, 5 minutes for instructions, 10 minutes for the experiment, and a 30-minute break.

This 2-session, within-participant study was conducted in Japan and enrolled 40 healthy young individuals (aged 18‐30 y). After written informed consent and instructions (10 min), participants were video-recorded while mimicking neutral, positive, and negative facial expressions (0.5 min each), followed by a 5-min task in which both participants and the researcher judged how well the AI-based valence classification of each facial expression matched the emotion conveyed. After a 30-minute break, the 3 mimicry blocks were repeated in a second session. The total time per participant was approximately 50 minutes.

Participants first completed a brief warm-up in which they practiced producing each target facial expression (positive, neutral, and negative) for approximately 10 seconds per expression, under the researcher’s guidance, to familiarize themselves with the procedure. The researcher then checked camera framing and instructed participants to adjust posture and face position to ensure consistent alignment for video recording. In the first phase, the participants were asked to display neutral (ie, normal), positive (ie, happy), and negative (eg, sad, frustrated, disgusted) facial expressions, each for 30 seconds, directed at the PC camera. The participants were not shown the results of the Kokoro Sensor analysis during this phase.

Additionally, both the participants and a researcher (SY) used a 4-point Likert-type scale with predefined anchors to evaluate the degree of agreement between the Kokoro Sensor’s emotion detection and the participants’ self-reported emotional states. Importantly, the objective of this study was not to establish whether the Kokoro Sensor adheres to a FACS-based microexpression taxonomy but instead to determine whether it can detect broad emotional valence of the kind typically evaluated in clinical contexts. Consequently, the human rating task was restricted to positive, neutral, and negative valence, which can be judged reliably without specialized FACS training. The rater’s role, therefore, centered on global valence matching rather than fine-grained action-unit coding, which we considered an appropriate level of expertise for the present aims.

To assess agreement, both participants and the researcher responded to specific prompts using a 4-point Likert scale (1=strongly disagree, 2=disagree, 3=agree, 4=strongly agree). Participants evaluated the prompt: “How well does the Kokoro Sensor’s result match the emotion you intended to express in this block?” In contrast, the researcher evaluated the prompt: “How well does the Kokoro Sensor’s result match the target emotion instructed for this block?” Notably, a neutral option was omitted to encourage decisive responses regarding the congruence between the sensor’s detections and participants’ emotions. This process was then repeated after a 30-minute interval to investigate reliability.

### Statistical Analyses: Concurrent Validity Relative to Human Ratings

As described above in the study procedure, concurrent validity was assessed by comparing the Kokoro Sensor’s AI-estimated valence classifications (neutral, positive, and negative) with human ratings. For each experimental block, we computed the proportions of frames classified as positive, neutral, and negative. Each block was labeled by the modal valence (ie, the category with the largest frame proportion). Block-level frame consistency was defined as the modal-valence proportion (ie, the maximum of the 3 frame proportions). Blocks with frame consistency of 75% or higher were classified as “stable,” and those with frame consistency of less than 75% as “unstable.” All blocks were retained; when frame consistency was less than 75%, the block was still labeled using the modal valence but interpreted as low consistency.

Concurrent validity was evaluated separately against participants’ ratings and the researcher’s ratings as 2 independent reference standards. When participants and the researcher disagreed for the same block, no adjudication (eg, consensus, averaging, or exclusion) was performed; both ratings were retained and analyzed independently, and interrater agreement between the participant and researcher ratings was not calculated. Following the content validity index (CVI) approach [28], perfect agreement (%) for each valence category was calculated as the proportion of ratings scored 3 or 4 on the 4-point Likert-type scale and was computed separately for participants and the researcher. In this study, a CVI value of 0.75 or higher was considered an acceptable level of concurrent validity.

Although the CVI is widely used, it does not account for inflated values resulting from chance agreement. To address this limitation, the weighted κ statistic [29] was calculated for agreement between the Kokoro Sensor’s AI-estimated valence classifications and the target-posed valence condition to provide a more robust test of overall agreement. This analysis evaluated agreement against the intended experimental condition rather than the Likert-based human ratings. Notably, the CVI was calculated against human perceived valence ratings, whereas the weighted κ was calculated against the intended posed-valence condition, therefore addressing complementary but non-identical reference standards. Additionally, κ coefficients were calculated separately for distinguishing positive from neutral valence and for distinguishing negative from neutral valence.

### Test-Retest Reliability

We assessed the test-retest reliability of the Kokoro Sensor by comparing its valence classifications between session 1 and session 2, which were separated by a 30-minute interval. In both sessions, the same experimental procedures (3 emotional-expression mimicry blocks) were administered. For each 30-second block, framewise predictions were summarized as the proportions of frames classified as positive, neutral, and negative valence. Each block was labeled by the modal valence (ie, the category with the largest frame proportion). Block-level frame consistency was defined as the modal-valence proportion (maximum frame proportion). The same 75% threshold was used to stratify analyses by block-level consistency, classifying blocks as stable (≥75%) or unstable (<75%), for sensitivity analyses.

For statistical analysis, we computed both weighted κ and unweighted κ coefficients with 95% CI. Test-retest reliability was quantified using the weighted κ for overall agreement across the 3 valence categories (positive, neutral, and negative) and unweighted κ for pairwise contrasts (distinguishing positive from neutral and distinguishing negative from neutral). All analyses were conducted in R (version 4.3.1, “Beagle Scouts”). κ values were calculated using the kappa.stat function (Aoki, Gunma University), implemented via the *vcd* package together with supplemental functions sourced from the publicly available script repository [30]. Agreement outcomes for each valence category were organized into cross-classification tables for κ calculation.

### Interpretation of the κ Coefficient

In this study, the interpretation of the weighted κ coefficients was based on standard thresholds for domain-specific judgments: values less than 0.20 were classified as indicating poor agreement, 0.21 to 0.40 or less as fair agreement, 0.41 to 0.60 or less as moderate agreement, 0.61 to 0.80 or less as substantial agreement, and greater than 0.81 as almost perfect agreement [29].

### Sensitivity Analyses

The robustness of the findings was assessed in 3 ways. First, to examine whether agreement differed by within-block valence stability, analyses were stratified by block-level consistency, defined as the modal-valence proportion (ie, the maximum proportion of frames assigned to a single valence within a block), with blocks classified as stable blocks (consistency ≥75%) or unstable blocks (consistency <75%). Second, to examine sensitivity to the definition of “high-consistency” blocks, we repeated the κ-based analyses after restricting the dataset to blocks whose modal-valence proportion met alternative frame consistency thresholds (≥60% and ≥90%). Third, analyses were repeated after stratification by sex.

All analyses were performed using R (version 4.3.1; available at [31]). The level of statistical significance was set at *P*<.05 (2-tailed).

### Ethical Considerations

Written informed consent was obtained from each participant prior to their involvement in the study. This study adhered to the ethical principles outlined in the Declaration of Helsinki and was approved by the ethics review board of the School of Allied Health Sciences at Kitasato University (approval number 2023‐032). All collected data were anonymized before analysis to ensure participant confidentiality and privacy. All study participants were compensated with a QUO card valued at 1000 JPY (approximately US $7) as an honorarium.

## Results

The characteristics of the participants (N=40; n=24, 60% male; median age: 21.0, IQR 21.0‐22.0 y) are shown in Table 1. In addition, POMS-SF descriptive statistics are summarized in Table 2. T-scores were generally centered on the normative mean, with few elevations of 70 or higher across subscales; positive dimensions were higher (Vigor-Activity mean: 55.0, SD 10.3; F mean: 58.7, SD 9.5). No participant met the exclusion threshold (TMD ≥70); all 40 were included in the analyses.

**Table 1.** Characteristics of the participants (N=40).

Characteristics	Values
Age (y), median (IQR)	21.0 (21.0‐22.0)
Sex, n (%)	
Male	24 (60)
Female	16 (40)

**Table 2.** Profile of Mood States–Short Form (POMS-SF) score descriptors (N=40).

Scale	Mean (SD)	Median (IQR)	Min-max T-score	T-score ≥70, n (%)
TMD^a^ score	46.1 (9.6)	42.5 (39.5-50)	31-66	0 (0)
AH^b^ score	43.5 (7.2)	41.0 (38.0-46.0)	36-64	0 (0)
CB^c^ score	50.1 (11.0)	46.5 (41.0-59.0)	36-76	2 (5)
DD^d^ score	48.5 (8.7)	45.0 (42.0-54.0)	39-68	0 (0)
FI^e^ score	46.5 (9.6)	44.0 (41.0-51)	33-73	1 (2.5)
TA^f^ score	49.0 (9.9)	47.0 (42.0-57)	35-71	1 (2.5)
VA^g^ score	55.0 (10.3)	55.0 (46.5-62.0)	36-74	3 (7.5)
F^h^ score^i^	58.7 (9.5)	59.5 (53.0-66.0)	38-78	4 (10)

a TMD: Total Mood Disturbance.

b AH: Anger-Hostility.

c CB: Confusion-Bewilderment.

d DD: Depression-Dejection.

e FI: Fatigue-Inertia.

f TA: Tension-Anxiety.

g VA: Vigor-Activity.

h F: Friendliness.

i Friendliness is not included in TMD.

The success rate for meeting the frame consistency criterion, defined as 75% or higher of frames classified under a single valence within a 30-second block, was 86.7% (104/120). Accordingly, 13.3% (16/120) of the blocks were classified as low consistency at the 75% threshold. In sensitivity analyses, the success rates were 93.3% (112/120) at the 60% threshold and 81.7% (98/120) at the 90% threshold.

Table 3 shows the CVI values, while Table 4 presents the weighted κ and κ coefficients for the concurrent validity assessments. As the “percentage of perfect agreement (%)” corresponds to the proportion of ratings scored 3 or greater on the Likert scale, these values are reported as CVI in Table 3. Among the participants, the CVI values for positive, neutral, and negative expressions were 95%, 98%, and 85%, respectively; for the researcher, the corresponding CVI values were 100%, 100%, and 70%. The overall weighted κ coefficient was 0.55 (95% CI 0.44‐0.67), indicating moderate agreement. For distinguishing positive from neutral expressions, the κ coefficient was 0.83 (95% CI 0.70‐0.95), indicating almost perfect agreement. For distinguishing negative from neutral expressions, the κ coefficient was 0.15 (95% CI –0.07 to 0.37), indicating no statistical significance.

**Table 3.** Concurrent validity for positive, neutral, and negative expressions^a^.

Expression	CVI^b^ (%)—participants	CVI (%)—researcher
Positive	95	100
Neutral	98	100
Negative	85	70

a For each expression category, the content validity index was calculated as the proportion of ratings scored 3 (agree) or 4 (strongly agree) on a 4-point Likert-type scale, divided by the total number of ratings, and expressed as a percentage. For example, for positive expressions rated by participants, if 38 out of 40 ratings were 3 or 4, then content validity index was 95%.

b CVI: content validity index.

**Table 4.** Kappa coefficients for concurrent validity assessments across expression categories^a^.

Concurrent validity	κ coefficient	95% CI	
		Lower	Upper
Overall valence classification^b^	0.55	0.44	0.67
Distinguishing positive from neutral	0.83	0.70	0.95
Distinguishing negative from neutral	0.15	–0.07	0.37

a For each 30-second block, framewise classifications were summarized as the proportions of frames classified as positive, neutral, and negative. Blocks were retained regardless of frame consistency and were labeled using the modal valence (largest frame proportion). Frame consistency was defined as the modal-valence proportion (maximum frame proportion); the 75% threshold was used to classify blocks as stable (≥75%) or unstable (<75%) in stratified sensitivity analyses (Tables S1-S3 in Multimedia Appendix 1).

b Weighted κ coefficient.

Table 5 presents the results of the test-retest reliability analysis. Test-retest agreement for distinguishing positive from neutral expressions was satisfactory, whereas agreement for distinguishing negative from neutral expressions was inadequate. The overall weighted κ coefficient was 0.66 (95% CI 0.55‐0.76), with κ coefficients of 0.85 (95% CI 0.73‐0.97) for distinguishing positive from neutral expressions and 0.36 (95% CI 0.16‐0.57) for distinguishing negative from neutral expressions.

Sensitivity analyses were conducted to assess robustness. First, analyses were stratified by block-level consistency (consistency ≥75% or <75%). In stable blocks (consistency ≥75%), κ-based concurrent validity estimates and test-retest reliability estimates were comparable to or slightly higher than those in the main analyses (Tables S1-S3 in Multimedia Appendix 1). In unstable blocks (consistency <75%), the overall concurrent validity remained relatively preserved (Table S2 in Multimedia Appendix 1), whereas κ-based contrasts showed lower agreement and less precise estimates (Tables S2 and S3 in Multimedia Appendix 1), likely due to sparse cell counts. Second, the results were materially unchanged when alternative thresholds of 60% and 90% were applied (Tables S4-S6 for concurrent validity and Tables S7-S9 for test-retest reliability in Multimedia Appendix 1). Third, analyses stratified by sex showed broadly similar patterns, except for female participants in the “distinguishing negative from neutral” condition under alternative thresholds (Tables S7-S9 in Multimedia Appendix 1).

**Table 5.** Test-retest reliability analysis: κ coefficients for positive, neutral, and negative expressions^a^.

Test-retest reliability	κ coefficient	95% CI	
		Lower	Upper
Overall valence classification^b^	0.66	0.55	0.76
Distinguishing positive from neutral	0.85	0.73	0.97
Distinguishing negative from neutral	0.36	0.16	0.57

a For each 30-second block, framewise classifications were summarized as the proportions of frames classified as positive, neutral, and negative. Blocks were retained regardless of frame consistency and were labeled using the modal valence (largest frame proportion). Frame consistency was defined as the modal-valence proportion (maximum frame proportion); the 75% threshold was used to classify blocks as stable (≥75%) or unstable (<75%) in stratified sensitivity analyses (Tables S1-S3 in Multimedia Appendix 1). The results of the analyses are shown as κ coefficients and corresponding 95% CI.

b Weighted κ coefficient.

## Discussion

### Principal Findings

This study assessed the concurrent validity and test-retest reliability of the Kokoro Sensor, an AI-based tool designed to detect emotional states based on facial expressions. The findings indicated that concurrent validity and reliability were satisfactory for overall valence classification and for distinguishing positive from neutral expressions. On the other hand, the concurrent validity for distinguishing negative from neutral expressions was not statistically significant, and limited reliability was observed. These findings represent a first step in exploring the potential clinical applications of this tool.

### Interpretation of the Findings for Distinguishing Positive From Neutral Findings

These findings showed satisfactory concurrent validity and test-retest reliability for distinguishing positive from neutral expressions. The CVI for distinguishing positive from neutral expressions exceeded both the concurrent validity benchmark set in our study (CVI ≥0.75) and that set in previous studies (CVI >0.78) [32,33], supporting adequate concurrent validity. Additionally, the κ coefficient for reliability was 0.85 (95% CI 0.73‐0.97), indicating almost perfect agreement, as a κ coefficient 0.81 or higher is generally considered indicative of this level of agreement [29].

This result suggests a high level of consistency in distinguishing between positive and neutral expressions. In support of these findings, previous studies have shown that distinguishing between positive and neutral expressions is generally clear and associated with minimal ambiguity, which facilitates consistent interpretation and AI processing [34-36]. For example, a previous study suggested that positive expressions such as smiles are generally more consistent because they involve clear changes in specific facial areas (eg, the mouth), which makes them easier for AI to recognize [36]. Another previous study reported that AI systems generally trained on datasets often learn positive expressions more extensively because of their higher prevalence in daily life, resulting in improved processing accuracy for positive emotions [37]. This phenomenon may also apply to the Kokoro Sensor dataset.

### Challenges in Distinguishing Negative From Neutral Expressions

By contrast, the CVI for negative expressions was 0.85 for participants and 0.70 for the researcher, indicating that the participants’ ratings met the concurrent validity benchmark set in our study (CVI≥0.75), whereas the researcher’s ratings for negative expressions failed to meet this benchmark. In addition, the ability to distinguish between negative and neutral valence was not statistically significant, as the κ for concurrent validity was 0.15 (95% CI –0.07 to 0.37). Additionally, the reliability of these distinctions showed limited agreement, with a κ of 0.36 (95% CI 0.16‐0.57).

Notably, our findings suggested that the inconsistency between the CVI of negative valence and the κ coefficient for distinguishing negative from neutral may be attributable to differences in the comparator and agreement metrics. The CVI indicates the degree of agreement between the results of the Kokoro Sensor and the participants’ and researchers’ judgments of negative valence, while the κ coefficient provides a chance-corrected index of agreement for distinguishing negative from neutral valence. Our findings suggest that although the Kokoro Sensor may estimate negative emotions from facial expressions, it might not adequately distinguish between negative and neutral expressions estimated from facial expressions.

There is 1 possible explanation for the difficulty in distinguishing between negative and neutral expressions. Negative facial expressions consist of smaller movement changes in facial expression muscle configurations [38], which makes them less recognizable than positive expressions. Consequently, the boundary between negative and neutral expressions is frequently less distinct than that between positive and neutral expressions [38], a challenge that may be further exacerbated by culturally shaped tendencies toward subdued or suppressed negative expressivity, particularly in East Asian populations. Furthermore, Affectiva [13] reports that its models are trained and tested on a global dataset of over 14 million videos collected from 90 countries but does not disclose detailed demographic information (eg, ethnicity and the proportion of East Asian faces). If East Asian populations are underrepresented compared to the intended deployment environment, a distribution shift between training and use populations could contribute to the reduced performance in the Japanese cohort, particularly for subtle negative facial expressions. This possibility is consistent with broader concerns that facial analysis performance may vary across demographic subgroups.

In addition, the study protocol itself may have contributed to reducing within-block consistency in framewise valence classifications. Participants were required to hold a posed facial expression for 30 seconds, and sustaining a static configuration for this duration may induce facial muscle fatigue and gradual, natural relaxation toward a more neutral state. Such sustained-posing requests may result in time-dependent changes in expression intensity or muscle activation, which could increase frame-to-frame variability within a block and thereby elevate the proportion of blocks that failed to meet the prespecified benchmark set in our study.

The observed reliability should likewise be interpreted in light of this study protocol. Participants were instructed to reproduce the same target facial expression across sessions. However, because the protocol relied on posed expressions, session-to-session differences in how individuals enacted the target expressions (eg, intensity or configuration when reproducing “sadness”) may have reduced κ even under stable sensor performance. The observed reliability likely reflects both sensor-related factors and within-participant inconsistency, which cannot be disentangled in this study design.

### Future Directions

These findings, along with previous consistent findings in both Western and Eastern contexts, emphasize the need for modifications based on new empirical evidence. Some previous studies in Western contexts have reported that emotional facial expressions generated based on scenarios (eg, “show the facial expressions you would typically display when experiencing the emotions triggered by the following situations”) are not always consistent with Ekman’s theory of prototypical expressions [39-41]. While the evidence is limited to Eastern contexts, 1 previous study [42] using Ekman-based analyses with FaceReader reported that emotions such as “happiness” and “surprise” are recognizable, whereas the recognition of other emotional expressions (eg, anger, disgust, fear, sadness) remains more difficult. Given that previous reports have investigated cultural differences in facial expressions between Western and Eastern contexts [18], and few studies have focused only on Eastern contexts, future research should investigate how cultural variations influence facial expression recognition and how these insights could be applied to improve AI systems.

### Clinical Applications

Our findings suggest that the Kokoro Sensor may have limited clinical utility for differentiating negative from neutral valence. The validity and reliability for estimating overall emotional valence and for distinguishing positive from neutral expressions were generally acceptable, indicating that the system may provide a useful indication of overall positive affect. Nevertheless, caution is warranted when using the Kokoro Sensor to differentiate negative from neutral valence, as its validity and reliability were weaker. Notably, the researcher’s CVI for negative expressions (CVI=0.70) fell below the prespecified benchmark (CVI ≥0.75). In particular, a κ value of 0.36 for distinguishing negative from neutral expressions indicates limited reliability for this clinically relevant contrast, which further constrains stand-alone clinical use.

From a psychometric perspective, κ values in the range of 0.40‐0.60 are typically interpreted as reflecting only moderate agreement, which is insufficient for stand-alone clinical decision-making. The κ value of 0.55 observed for overall valence classification supports the use of the Kokoro Sensor as an adjunctive or screening aid rather than as an independent diagnostic instrument. Although there is no generally accepted consensus on κ thresholds for AI-based facial-affect detection, many health care applications adopt κ 0.80 or higher as a benchmark for diagnostic deployment. Accordingly, substantial gains in accuracy and temporal stability would be necessary before the Kokoro Sensor could be considered suitable for routine clinical decision-making.

The low agreement for distinguishing negative from neutral valence (κ=0.15) further underscores an important limitation. When the sensor yields negative, ambiguous, or clinically incongruent outputs, clinicians should actively seek converging evidence from independent sources, such as structured observational scales, physiological indicators (eg, heart rate variability, actigraphy), or voice-based markers (eg, prosodic and other acoustic features).

### Limitations

This study has some limitations. First, the scenario-based induction of emotional facial expressions enabled a systematic examination across a wide range of emotions [40]. For this pilot study, we used posed mimicry rather than mood induction to ensure a feasible and standardized protocol for initial device evaluation. This approach allowed for tighter control over target expressions and reduced procedural variability across participants and sessions. However, it may limit generalizability to naturalistic affective states. Posed expressions, even when scenario-based, do not fully capture the nuanced, blended, and transient facial displays that emerge spontaneously in real-world clinical settings. Such settings involve greater individual variability in imagery capacity and deliberate emotion masking (eg, of pain or anxiety)[43], making posed expressions an imperfect surrogate and potentially overestimating performance. In addition, baseline POMS-SF scores indicated a relatively positive mood (Vigor-Activity T=55.0; Friendliness T=58.7), which may have made it more difficult for participants to authentically pose negative expressions (eg, reduced expressivity or emotion masking), potentially attenuating the negative-neutral contrast and thereby limiting the Kokoro Sensor’s ability to differentiate negative from neutral expressions. Moreover, our blocked design—using distinct 30-second segments per target emotion—likely reduced ambiguity by constraining participants to a single labeled affective state at a time. While this improved experimental control, it may have limited the occurrence of transitional, mixed, or low-intensity expressions typical in natural contexts, thereby introducing spectrum bias and inflating validity metrics. Future studies should incorporate validated mood-induction paradigms (eg, standardized film clips or scripted scenarios) within clinical environments to enhance ecological validity [44]. Second, the validity of the Kokoro Sensor in distinguishing negative from neutral expressions is uncertain, and any inferences about its detection of negative valence should be made cautiously. The κ coefficient for this contrast was low and nonsignificant (κ=0.15; 95% CI –0.07 to 0.37), suggesting that the system failed to demonstrate reliable discrimination between negative and neutral valence. The wide confidence interval likely reflects a combination of the modest sample size and the intrinsic challenge of separating subtle negative expressions from neutral ones. Due to the use of an overall 3×3 weighted κ test in the prior sample size determination, this study may have had limited power/precision for binary contrasts (eg, negative vs neutral). Consistent with the low κ test values and wide confidence intervals observed for these contrasts, nonsignificant results should be interpreted with caution, as the possibility of a type 2 error cannot be excluded. Although a post hoc sex-stratified analysis suggested minimal sex differences overall, the apparent deviation observed among women in the “negative from neutral” condition should be interpreted cautiously because sex-specific hypotheses were not prespecified, and subgroup samples were small with imprecise estimates. Third, a subset of blocks did not meet the prespecified frame-consistency threshold (ie, <75% of frames classified under a single valence). For these low-consistency blocks, block-level labels were assigned using the modal valence, which may be less reliable than labels derived from high-consistency segments. Therefore, aggregating low-consistency blocks with high-consistency blocks in the main analyses may obscure potentially important differences in Kokoro Sensor performance between more stable and less stable conditions, effectively averaging across heterogeneous performance conditions. Although stratified sensitivity analyses by block-level consistency were conducted (Tables S1-S3 in Multimedia Appendix 1), estimates in the low-consistency subgroup should be interpreted cautiously due to sparse cell counts and reduced precision. Fourth, we assessed test-retest reliability over a 30-minute interval, which is brief and may allow memory or carryover effects. Longer intervals (eg, 24‐48 h) and multisession designs are needed to establish temporal stability more robustly. Fifth, human ratings relied on participants and a researcher using predefined anchors; this precluded estimation of interrater reliability. Additionally, because the 4-point Likert scale excluded a neutral midpoint by design, raters were compelled to choose a valence category when the AI valence classification was ambiguous. Such forced-choice responding may have biased responses toward the endorsement of a category and may have artificially increased observed agreement. Sixth, because the researcher provided instructions and was present during the sessions, the researcher’s ratings were not blinded to the target emotion or condition in each block. This lack of blinding may have introduced observer bias into the researcher-rated dataset, potentially resulting in an overestimation of agreement estimates. Finally, our sample consisted of young Japanese adults, limiting generalizability to other age groups and to clinical populations where facial morphology (eg, wrinkles) or comorbid conditions (eg, facial palsy) may affect AI performance. Cross-cultural differences in expression production, such as those between Eastern and Western populations, may further influence recognition accuracy [18]. Future studies should recruit broader samples that vary in age, culture, and clinical status.

### Conclusion

The findings of this study suggest that the Kokoro Sensor may be useful for identifying positive affect, given its acceptable concurrent validity for overall valence estimation and high agreement for distinguishing positive from neutral expressions. However, the prespecified benchmark for concurrent validity was not met for negative expressions based on the researcher’s ratings, and agreement for distinguishing negative from neutral expressions was limited, which may constrain its clinical utility for detecting negative affect. Therefore, the Kokoro Sensor may be best used as an assistive tool rather than a stand-alone method in clinical settings.

## Supplementary material

10.2196/81868 Multimedia Appendix 1 Sensitivity analyses.

## Abbreviations

AI: artificial intelligence
COSMIN: Consensus-based Standards for the Selection of Health Measurement Instruments
CVI: content validity index
FACS: facial action coding system
POMS-SF: Japanese Profile of Mood States–Short Form
TMD: Total Mood Disturbance
