# Supplementary material for: Facial Expression–Based Evaluation of the Emotion Estimation Software Kokoro Sensor in Healthy Individuals: Validation and Reliability Pilot Study
Source: JMIR AI. 2026 Feb 26;5:e81868. doi: 10.2196/81868 (PMC12945095; doi:10.2196/81868)
Supplement: Multimedia Appendix 1 [file ai-v5-e81868-s001.docx]

**Table S1. Sensitivity analysis of concurrent validity for positive, neutral, and negative expressions in stable (consistency ≥ 75%) and unstable (consistency < 75%) experimental blocks**

| Expression | CVI (%) – Participants | CVI (%) –Researcher |
| --- | --- | --- |
| **Blocks consistency ≥ 75%** |  |  |
| Positive | 94.3 | 100 |
| Neutral | 97.5 | 100 |
| Negative | 82.8 | 65.5 |
| **Blocks consistency < 75%** |  |  |
| Positive | 100 | 100 |
| Neutral | Not estimable (n=0) | Not estimable (n=0) |
| Negative | 90.9 | 81.8 |

**Note**: For each expression category, the Content Validity Index (CVI) was calculated as the proportion of ratings scored 3 (agree) or 4 (strongly agree) on a four-point Likert-type scale, divided by the total number of ratings, and expressed as a percentage. For example, for positive expressions rated by participants, if 38 out of 40 ratings were 3 or 4, then CVI = 95%. In the unstable subgroup, no blocks were labeled as neutral (n = 0); therefore, the CVI for neutral expressions could not be estimated.

**Table S2. Sensitivity analysis of agreement metrics in stable (consistency ≥ 75%) and unstable (consistency < 75%) experimental blocks**

|  | κ coefficient | 95% CI | |
| --- | --- | --- | --- |
|  |  | Lower | Upper |
| **Concurrent validity** |  |  |  |
| **Blocks consistency ≥ 75%** |  |  |  |
| Overall valence classification* | 0.67 | 0.55 | 0.78 |
| Distinguishing positive from neutral | 0.95 | 0.87 | 1.02 |
| Distinguishing negative from neutral | 0.24 | -0.02 | 0.50 |
| **Blocks consistency < 75%** |  |  |  |
| Overall valence classification* | 0.66 | 0.42 | 0.91 |
| Distinguishing positive from neutral | 0.57 | -0.20 | 1.34 |
| Distinguishing negative from neutral | 0.00 | -0.78 | 0.78 |

Note: For each 30-second block, framewise classifications were summarized as the proportions of frames classified as positive, neutral, and negative. The results of the analyses are shown as κ coefficients and corresponding 95% confidence intervals (CIs).

* Weighted κ coefficient

**Table S3. Sensitivity analysis of test–retest reliability for agreement metrics in stable (consistency ≥ 75%) and unstable (consistency < 75%) experimental blocks**

|  | κ coefficient | 95% CI | |
| --- | --- | --- | --- |
|  |  | Lower | Upper |
| **Test-retest reliability** |  |  |  |
| **Blocks consistency ≥ 75%** |  |  |  |
| Overall valence classification* | 0.70 | 0.60 | 0.81 |
| Distinguishing positive from neutral | 0.92 | 0.83 | 1.01 |
| Distinguishing negative from neutral | 0.42 | 0.20 | 0.63 |
| **Blocks consistency < 75%** |  |  |  |
| Overall valence classification* | 0.26 | -0.09 | 0.60 |
| Distinguishing positive from neutral | 0.00 | 0.00 | 0.00 |
| Distinguishing negative from neutral | 0.00 | -0.98 | 0.98 |

Note: For each 30-second block, framewise classifications were summarized as the proportions of frames classified as positive, neutral, and negative. The results of the analyses are shown as κ coefficients and corresponding 95% confidence intervals (CIs).

* Weighted κ coefficient

**Table S4. Kappa Coefficients (≥ 75% Valence Threshold) for Concurrent Validity Across Expression Categories by Sex**

|  | κ coefficient | 95% CI | |
| --- | --- | --- | --- |
|  |  | Lower | Upper |
| **Concurrent validity** |  |  |  |
| Overall valence classification* |  |  |  |
| Male | 0.63 | 0.50 | 0.76 |
| Female | 0.43 | 0.24 | 0.63 |
| Distinguishing positive from neutral |  |  |  |
| Male | 0.88 | 0.74 | 1.01 |
| Female | 0.75 | 0.52 | 0.98 |
| Distinguishing negative from neutral |  |  |  |
| Male | 0.25 | -0.02 | 0.52 |
| Female | 0.00 | -0.36 | 0.36 |

Note: If the proportion of frames with a positive, negative, or neutral valence within a 30-second interval exceeded 75%, the experimental condition was classified under that corresponding valence. The results of the analyses are shown as κ coefficients and corresponding 95% confidence intervals (CIs).

* Weighted κ coefficient

**Table S5. Kappa Coefficients (≥ 90% Valence Threshold) for Concurrent Validity Across Expression Categories in the Overall Sample and by Sex**

|  | κ coefficient | 95% CI | |
| --- | --- | --- | --- |
|  |  | Lower | Upper |
| **Concurrent validity** |  |  |  |
| Overall valence classification* | 0.52 | 0.40 | 0.63 |
| Male | 0.61 | 0.48 | 0.75 |
| Female | 0.38 | 0.18 | 0.58 |
| Distinguishing positive from neutral | 0.75 | 0.61 | 0.89 |
| Male | 0.83 | 0.68 | 0.99 |
| Female | 0.63 | 0.35 | 0.90 |
| Distinguishing negative from neutral | 0.15 | -0.07 | 0.37 |
| Male | 0.25 | -0.02 | 0.52 |
| Female | 0.00 | -0.35 | 0.35 |

Note: If the proportion of frames with a positive, negative, or neutral valence within a 30-second interval exceeded 90%, the experimental condition was classified under that corresponding valence. The results of the analyses are shown as κ coefficients and corresponding 95% confidence intervals (CIs).

* Weighted κ coefficient

**Table S6. Kappa Coefficients (≥ 60% Valence Threshold) for Concurrent Validity Across Expression Categories in the Overall Sample and by Sex**

|  | κ coefficient | 95% CI | |
| --- | --- | --- | --- |
|  |  | Lower | Upper |
| **Concurrent validity** |  |  |  |
| Overall valence classification* | 0.64 | 0.53 | 0.74 |
| Male | 0.63 | 0.50 | 0.76 |
| Female | 0.43 | 0.24 | 0.63 |
| Distinguishing positive from neutral | 0.88 | 0.77 | 0.98 |
| Male | 0.96 | 0.88 | 1.04 |
| Female | 0.75 | 0.52 | 0.98 |
| Distinguishing negative from neutral | 0.28 | 0.07 | 0.49 |
| Male | 0.42 | 0.16 | 0.67 |
| Female | 0.07 | -0.29 | 0.42 |

Note: If the proportion of frames with a positive, negative, or neutral valence within a 30-second interval exceeded 60%, the experimental condition was classified under that corresponding valence. The results of the analyses are shown as κ coefficients and corresponding 95% confidence intervals (CIs).

* Weighted κ coefficient

**Table S7. Kappa Coefficients (≥ 75% Valence Threshold) for Test–retest reliability Across Expression Categories by sex**

|  | κ coefficient | 95% CI | |
| --- | --- | --- | --- |
|  |  | Lower | Upper |
| Test–retest reliability |  |  |  |
| Overall valence classification* |  |  |  |
| Male | 0.73 | 0.60 | 0.86 |
| Female | 0.54 | 0.37 | 0.72 |
| Distinguishing positive from neutral |  |  |  |
| Male | 0.88 | 0.74 | 1.01 |
| Female | 0.81 | 0.61 | 1.01 |
| Distinguishing negative from neutral |  |  |  |
| Male | 0.53 | 0.28 | 0.77 |
| Female | 0.13 | -0.22 | 0.47 |

Note: If the proportion of frames with a positive, negative, or neutral valence within a 30-second interval exceeded 75%, the experimental condition was classified under that corresponding valence. The results of the analyses are shown as κ coefficients and corresponding 95% confidence intervals (CIs).

* Weighted κ coefficient

**Table S8. Kappa Coefficients (≥ 90% Valence Threshold) for Test–retest reliability Across Expression Categories in the Overall Sample and by Sex**

|  | κ coefficient | 95% CI | |
| --- | --- | --- | --- |
|  |  | Lower | Upper |
| Test–retest reliability |  |  |  |
| Overall valence classification* | 0.55 | 0.44 | 0.66 |
| Male | 0.62 | 0.47 | 0.76 |
| Female | 0.43 | 0.25 | 0.61 |
| Distinguishing positive from neutral | 0.73 | 0.57 | 0.88 |
| Male | 0.83 | 0.68 | 0.99 |
| Female | 0.56 | 0.28 | 0.85 |
| Distinguishing negative from neutral | 0.23 | 0.01 | 0.44 |
| Male | 0.31 | 0.03 | 0.58 |
| Female | 0.12 | -0.22 | 0.46 |

Note: If the proportion of frames with a positive, negative, or neutral valence within a 30-second interval exceeded 90%, the experimental condition was classified under that corresponding valence. The results of the analyses are shown as κ coefficients and corresponding 95% confidence intervals (CIs).

* Weighted κ coefficient

**Table S9. Kappa Coefficients (≥ 60% Valence Threshold) for Test–retest reliability Across Expression Categories in the Overall Sample and by Sex**

|  | κ coefficient | 95% CI | |
| --- | --- | --- | --- |
|  |  | Lower | Upper |
| Test–retest reliability |  |  |  |
| Overall valence classification* | 0.67 | 0.57 | 0.77 |
| Male | 0.73 | 0.60 | 0.86 |
| Female | 0.54 | 0.37 | 0.72 |
| Distinguishing positive from neutral | 0.85 | 0.73 | 0.97 |
| Male | 0.88 | 0.74 | 1.01 |
| Female | 0.81 | 0.61 | 1.01 |
| Distinguishing negative from neutral | 0.39 | 0.18 | 0.59 |
| Male | 0.60 | 0.37 | 0.83 |
| Female | 0.13 | -0.22 | 0.47 |

Note: If the proportion of frames with a positive, negative, or neutral valence within a 30-second interval exceeded 60%, the experimental condition was classified under that corresponding valence. The results of the analyses are shown as κ coefficients and corresponding 95% confidence intervals (CIs).

* Weighted κ coefficient
